# Hypofractionated Palliative Radiotherapy for Relapsed and Refractory High-Risk Neuroblastoma †

**DOI:** 10.3390/curroncol32030124

**Published:** 2025-02-22

**Authors:** Ellery Koelker-Wolfe, Karen J. Marcus, Steven G. DuBois, Paul J. Catalano, Suzanne Shusterman, Myrsini Ioakeim-Ioannidou, Hesham Elhalawani, Torunn I. Yock, Shannon M. MacDonald, Daphne A. Haas-Kogan, Kevin X. Liu

**Affiliations:** 1 Department of Radiation Oncology, Brigham and Women’s Hospital, Dana-Farber Cancer Institute, Boston Children’s Hospital, Harvard Medical School, Boston, MA 02115, USA; ekoelkerwolfe@hms.harvard.edu (E.K.-W.); kmarcus@mgb.org (K.J.M.); helhalawani@bwh.harvard.edu (H.E.); dhaas-kogan@bwh.harvard.edu (D.A.H.-K.); 2 Department of Pediatrics, Dana-Farber/Boston Children’s Cancer and Blood Disorders Center, Boston, MA 02115, USA; steven_dubois@dfci.harvard.edu (S.G.D.); suzanne_shusterman@dfci.harvard.edu (S.S.); 3 Department of Data Science, Dana-Farber Cancer Institute, Boston, MA 02215, USA; pcata@ds.dfci.harvard.edu; 4 Department of Radiation Oncology, Massachusetts General Hospital, Harvard Medical School, Boston, MA 02115, USA; mioakeimioannidou@mgh.harvard.edu (M.I.-I.); tyock@mgh.harvard.edu (T.I.Y.); smacdonald@mgh.harvard.edu (S.M.M.)

**Keywords:** hypofractionated radiotherapy, palliative radiotherapy, metastases, pain

## Abstract

**Introduction:** While palliative radiotherapy (RT) is frequently used in the management of relapsed/refractory high-risk neuroblastoma (HR-NBL); outcomes after palliative hypofractionated RT (hypo-RT) remain poorly characterized. **Methods**: We conducted a multi-institutional retrospective study of 38 patients who were diagnosed with HR-NBL between 1997 and 2021 and received palliative RT. Conventional RT (conv-RT) and hypo-RT were defined as palliative treatment courses using dose ≤2 or >2 Gy per fraction, respectively. The primary outcome was cumulative incidence of in-field progression using Gray’s test. Univariate analyses were performed using the Cox proportional hazards model. **Results**: When analyzing by first course of palliative RT, 16 patients received conventionally fractionated RT (43%) and 21 received hypo-RT (57%). Clinical characteristics were similar between the two groups. With a median follow-up of 10.3 months (range: 0.3–104.0), the cumulative incidence of in-field progression was not statistically significantly different between hypo-RT and conv-RT (30% vs. 20% at 10 months; *p* = 0.80). Clinical response, defined as symptomatic improvement or decrease in the size of the lesion, was not statistically different between the two groups (92% conv-RT vs. 90% hypo-RT; *p* = 1.00). No grade ≥4 toxicities were observed. On univariate analysis, hypo-RT (HR 1.50; 95% CI 0.47–4.76; *p* = 0.493) was not statistically significantly associated with time to in-field progression, but *MYCN* amplification was associated with significantly longer time to in-field progression (HR: 0.20; 95% CI: 0.05–0.77; *p* = 0.020). **Conclusions**: We found no statistically significant difference in cumulative incidence of in-field progression and clinical outcomes between the conv-RT and hypo-RT groups. Palliative hypo-RT can be considered for relapsed/refractory HR-NBL, especially when shorter treatments may offer improved quality of life.

## 1. Introduction

Neuroblastoma arises from precursor cells of the sympathetic nervous system and is the most common extracranial solid tumor in children [1]. Patients with neuroblastoma are commonly considered high-risk if they are older than 18 months old with metastatic disease or have local disease with concerning tumor characteristics such as *MYCN* amplification [2]. For patients with high-risk neuroblastoma, approximately 50% of patients suffer disease relapse despite an aggressive multimodality treatment with chemotherapy, surgical resection, hematopoietic stem cell transplantation, radiotherapy, and immunotherapy [3,4,5,6,7]. In addition, 54 of 652 (8.3%) patients with high-risk neuroblastoma enrolled in the most recently completed Children’s Oncology Group (COG) study ANBL0532 had refractory or progressive disease before consolidation therapy [5]. Unfortunately, relapsed or refractory neuroblastoma portends a poor prognosis with a recent study showing 1- and 4-year progression-free survival rates of 21% ± 2% and 6% ± 1%, respectively, for patients enrolled in modern-era early-phase clinical trials for relapsed or refractory neuroblastoma [8].

Radiotherapy plays an important role in local control for patients with high-risk neuroblastoma. The optimal dose remains under investigation. The most recent prospective study, COG ANBL0532, showing that boost radiotherapy to gross residual disease does not improve local control [4]. In addition, palliative radiation therapy is frequently used in the management of painful or symptomatic metastases in patients with refractory or progressive neuroblastoma [9,10,11]. While various palliative fractionation regimens have been described for neuroblastoma, the utility of hypofractionated radiation therapy (hypo-RT) in the management of relapsed or refractory neuroblastoma remains poorly defined. Hypo-RT describes a treatment regimen that delivers larger doses of radiation in fewer fractions and over a shorter period of time compared to conventionally fractionated radiotherapy (conv-RT). Hypofractionated radiotherapy has the potential advantage of shorter treatment courses, reducing the need for sedation in pediatric patients and avoiding prolonged courses in the palliative setting. Recent findings by Sudmeier et al. suggest that the efficacy of hypo-RT for palliation in adults may be generalizable to pediatric patients [12]. Thus, we performed a multi-institutional retrospective analysis of patients with relapsed or refractory high-risk neuroblastoma to investigate clinical outcomes, including symptom relief and local control, after receipt of palliative hypo-RT compared to conv-RT.

## 2. Methods

The Dana-Farber Cancer Institute Institutional Review Board approved a retrospective study of 38 patients who were diagnosed with high-risk neuroblastoma between 1997 and 2021 and received palliative radiation therapy for relapsed or refractory disease at Brigham and Women’s Hospital and Massachusetts General Hospital. High-risk neuroblastoma was defined using COG guidelines as previously described [2]. Patients receiving radiation therapy for central nervous system and liver metastases were excluded as studies have found that conventionally fractionated radiation is standard for these patients and no patients with central nervous system or liver metastases received hypo-RT at our institutions [9,13].

We collected data regarding clinical and treatment characteristics, including sex, age at diagnosis, race/ethnicity, age at first course of treatment, progression of disease before palliative radiation therapy, site of disease, and course of chemotherapy received before, during, and after radiation therapy. Characteristics of radiation therapy, including total dose and dose per fraction, were recorded. Conventional RT (conv-RT) and hypo-RT were defined as palliative treatment courses using dose ≤2 or >2 Gy per fraction, respectively. Radiation biologically effective dose (BED) was calculated using an α/β of 10 for the tumor. Outcomes such as symptomatic relief, decrease in the size of the lesion by diagnostic imaging, dates and location of in-field and out-of-field progression, and date of death were collected. Follow-up time was defined from the start of radiation therapy. Toxicity was assessed by CTCAE v5.

Statistical analyses were performed using Stata 15 (Stata Corp, College Station, TX, USA) and R [14]. Fisher exact tests were used for the comparison of categorical variables. Mann–Whitney U test was used for comparison of quantitative variables. Survival analyses were performed using the Kaplan–Meier method and log-rank test. The cumulative incidence of in-field progression was analyzed using Gray’s test with death as a competing risk. Univariate analyses (UVA) were performed using Cox proportional-hazards regression with competing risks regression for stratified and clustered data with death as a competing risk. *p*-values < 0.05 were deemed as significant.

## 3. Results

Patient demographics and disease characteristics are summarized in Table 1. Sixteen patients received a first course of conv-RT (43%) and 21 received a first course of hypo-RT (57%). Patients who had further progression of disease before palliative radiation therapy were more likely to receive hypo-RT (52% vs. 13%, *p* = 0.017). Other factors, including receipt of palliative radiation to bony metastases; receipt of chemotherapy before, during, or after palliative radiation; and symptoms at the time of radiotherapy, did not differ between the cohorts receiving conv-RT and hypo-RT (Table 1).

**Figure 1:**
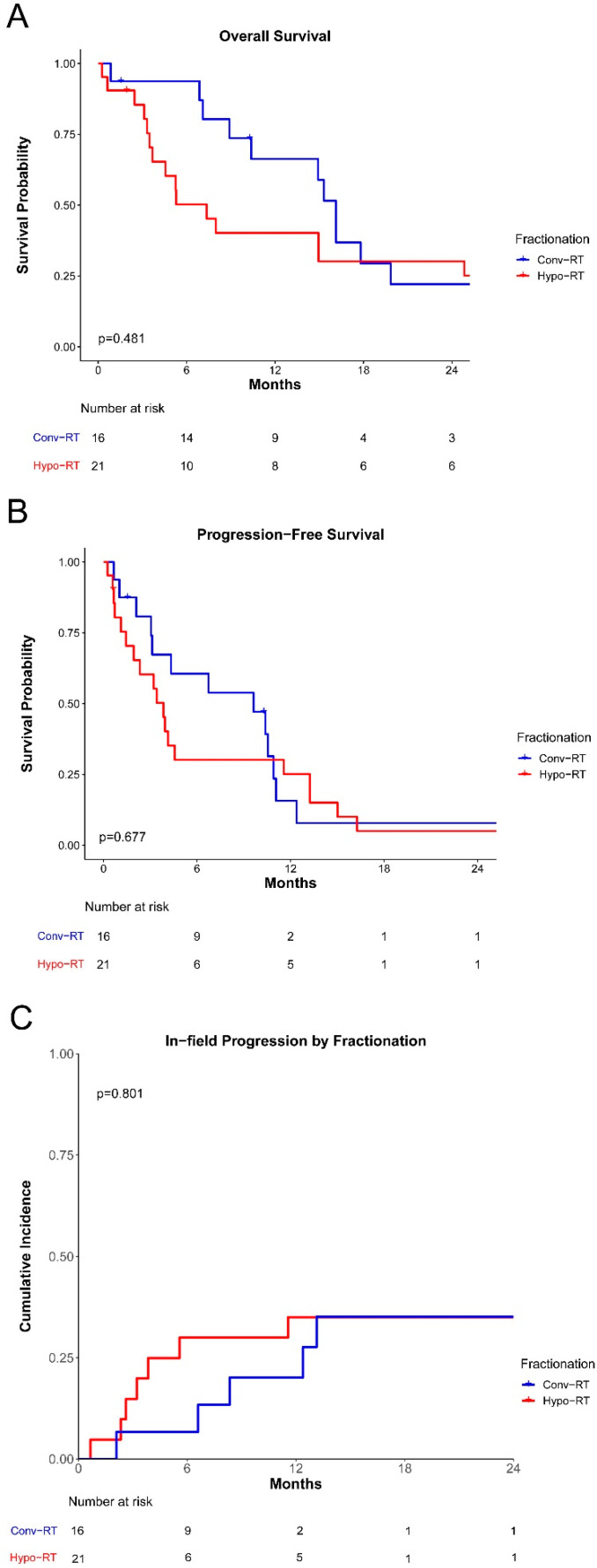
Survival and local progression after the first course of palliative radiotherapy. (**A**) Overall survival is stratified by conventionally fractionated and hypofractionated regimens. (**B**) Progression-free survival stratified by conventionally fractionated and hypofractionated regimens. (**C**) Cumulative incidence of in-field local progression stratified by conventionally fractionated and hypofractionated regimens.

We next analyzed whether clinical outcomes differed between patients receiving hypo-RT compared to those receiving conv-RT for their first course of palliative radiation. Median follow-up time was 10.3 months for all patients after the first course of radiotherapy (range: 0.3–104.0). Overall survival and progression-free survival were not significantly different among patients who received conv-RT vs. hypo-RT (Figure 1A,B). Median overall survival was 7.4 and 16.1 months for the hypo-RT and conv-RT groups, respectively, but this difference did not reach statistical significance (*p* = 0.48). The cumulative incidence of in-field progression at 10 months was not different between the conv-RT and hypo-RT groups (20% vs. 30%, *p* = 0.80, Figure 1C). After radiation therapy, patients who received conv-RT and hypo-RT had similar clinical benefits, defined as symptomatic relief or any decrease in the size of the lesion (92% vs. 90%, *p* = 1.00, Table 1).

We next performed univariate analyses of parameters that are associated with time to in-field local progression (Table 2). Further progression of disease before RT, tumor site (bony vs. soft tissue), receipt of chemotherapy before RT, receipt of chemotherapy during RT, and receipt of chemotherapy after RT were not significant predictors of time to in-field progression (Table 2). In addition, hypo-RT (Subhazard Ratio [SHR]: 1.17, 95% confidence interval [CI]: 0.39–3.58, *p* = 0.778) was not associated with time to in-field progression. Given that a prior study found that boost radiotherapy did not improve local control for patients with high-risk neuroblastoma and gross residual disease after resection [4], we studied whether patients receiving palliative radiation doses with BED ≥ 25.5 Gy, equivalent to 21.6 Gy in 12 fractions, had improved outcomes. We found that BED ≥ 25.5 Gy was not associated with improved local control (SHR: 1.39, 95% CI: 0.38–5.05, *p* = 0.618). *MYCN* amplification was associated with a significantly longer time to in-field progression (HR: 0.20, 95% CI: 0.05–0.77, *p* = 0.020). In our cohort, there was no difference in progression-free survival (*p* = 0.445) and overall survival (0.449) between patients with and without *MYCN* amplification (Supplemental Figures S1 and S2). *MYCN* amplification was also not associated with the use of systemic therapy before, during, or after radiation nor was it associated with the use of an anti-GD2 antibody as part of the systemic therapy regimen at first relapse (Supplemental Table S1).

Given many patients received multiple courses of palliative radiation, we also analyzed clinical outcomes comparing the use of hypo-RT and conv-RT in any course of palliative radiotherapy. Clinical characteristics for any courses of palliative radiation are summarized in Table 3. Patients receiving hypo-RT were significantly more likely to receive radiation doses with a BED ≥ 25.5 Gy (75% vs. 43%, *p* = 0.005). We found that there was a trend towards patients being more likely to have progression of disease before palliative radiotherapy in the hypo-RT group (37% vs. 17%, *p* = 0.056). In addition, there was a trend towards patients being more likely to receive chemotherapy after conventionally fractionated palliative radiation (83% vs. 63%, *p* = 0.056) and receive radiation to asymptomatic lesions in the conv-RT group (37% vs. 17%, *p* = 0.062). Other clinical characteristics, including receipt of chemotherapy before and during palliative radiation, were not significantly different between the conv-RT and hypo-RT groups (Table 3). The rate of complete response on imaging was not statistically significantly different between the conv-RT and hypo-RT groups (29% vs. 20%, *p* = 0.304). For lesions with imaging follow-up, the median time to best imaging response was 46.5 days (range: 3–324). The median time to best imaging response was not statistically significantly different between lesions receiving conv-RT (62 days [range: 7–324]) or hypo-RT (29 days [range: 9–295], *p* = 0.192). Grade ≥ 2 toxicities were limited in both the conv-RT and hypo-RT groups and were expected after specific tumor locations and radiation fields, such as esophagitis after radiation to the thoracic spine or mucositis after radiation to head and neck metastases (Supplemental Table S2). There is no statistically significant difference in rates of grade 2 or 3 toxicity between the two groups (Supplemental Table S2). No grade 4 or 5 toxicities were observed.

We also performed univariate analyses to investigate factors that predict locoregional progression for all courses of palliative radiotherapy (Table 4). We found that bony sites of radiotherapy were associated with a longer time to in-field progression on univariate analysis compared to soft tissue sites (HR: 0.43, 95% CI: 0.23–0.82, *p* = 0.010, Table 4). Of note, patients received imaging studies tailored to specific sites of disease, with soft tissue sites more likely to receive additional CT-based imaging in follow-up while bony sites were followed using other imaging modalities (Supplemental Table S3). In addition, there was a trend of shorter time to in-field progression in patients receiving chemotherapy after palliative radiation (SHR: 3.61, 95% CI: 0.99–13.0, *p* = 0.050). Importantly, time to in-field progression for all courses did not differ between the hypo-RT and conv-RT groups (SHR: 0.89, 95% CI: 0.40–1.96, *p* = 0.760).

## 4. Discussion

Previous studies have described the use of conv-RT for the palliation of high-risk neuroblastoma or have shown the efficacy of hypo-RT in a broad range of diagnoses, but none have compared outcomes after conv-RT and hypo-RT fractionation [9,10,11,12]. Given the palliative nature of hypo-RT, shorter treatment courses are preferable; it has remained unclear, however, whether more condensed fractionation schedules offer adequate disease control. Our study presents data from one of the largest cohorts of palliative radiotherapy in pediatric patients with relapsed or refractory high-risk neuroblastoma. We found no difference in cumulative incidence of in-field local progression or symptomatic benefit after hypo-RT compared to conv-RT in patients with relapsed or refractory high-risk neuroblastoma.

We observed a median overall survival of 7.4 and 16.1 months after the first course of palliative radiation for the hypo-RT and conv-RT groups, respectively, a finding that was not statistically significantly different between the two groups, perhaps due to a small sample size. The median overall survival in our cohort was longer than previously reported in similar studies [8,9,10,11], likely due to improvements in treatment options for relapsed or refractory high-risk neuroblastoma in recent decades [15].

Our findings are consistent with previous studies that support the use of palliative radiotherapy for bony and soft tissue metastases in neuroblastoma patients [9,10,11,12]. We found response rates (defined as either symptomatic benefit or decrease in the size of the lesion) of 92.3% and 90.0% in patients receiving either conv-RT or hypo-RT, respectively. A prior study by Caussa et al. reported an 84.2% response rate for soft tissue metastases [9]. We found no statistically significant difference in radiographic response rates on imaging between the conv-RT and hypo-RT groups, with 28.6% and 20.0% of patients demonstrating complete responses, respectively. These findings are consistent with the complete response rates of 4–42% that have been previously reported [9,11]. We found that the cumulative incidence of locoregional progression at 10 months was 20.1% and 30.0% for the conv-RT and hypo-RT groups, respectively. The group of patients receiving conv-RT is enriched for refractory rather than relapsed disease, which may account for the longer overall survival in this group. While overall survival was not different between the two groups, the rates of local progression are higher than previously reported by Paulino et al., who reported local control of >90% [11]. The difference between our study and the Paulino et al. report is likely due to differences in overall survival after palliative radiation; the much longer overall survival of our cohort, compared to the 2.5 months reported by Paulino et al. [11], likely allows more long-living patients in our cohort time to develop local progression.

Of those patients who developed local progression, many had bony sites of metastasis. Prior studies have suggested that palliative radiation to soft tissue or bony lesions can have response rates >50% and excellent local control rates >90% [9,10,11]. Our analysis of all courses of palliative RT found that bony sites were associated with a longer time to in-field progression compared to non-bony sites of metastasis. Previous studies found that irradiation of bony metastases can decrease the rate of recurrence at first relapse and even doses as low as those used for total body irradiation may be sufficient in preventing recurrence at some sites [16,17]. It is possible that bony metastases respond better compared to other sites of metastases and that lower doses may be sufficient for local disease control and symptom management, a topic that warrants further investigation [16,17]. It is also possible that progression at bony sites is more difficult to examine by conventional imaging and that may contribute to this observation. In addition, we found that patients who received chemotherapy after palliative radiation had a shorter time to in-field progression. This could be because patients receiving chemotherapy had more extensive disease and therefore were at greater risk of rapid in-field progression.

In our cohort, *MYCN* amplification was associated with a longer time to in-field progression after palliative radiotherapy. Prior studies have found that *MYCN* amplification is associated with an increased risk of local failure for patients with newly diagnosed high-risk neuroblastoma [18,19], this finding may represent a false positive given the small size of the cohort. If the finding is real and not an artifact of a small cohort, it may be due to selection bias. We did not find any association between *MYCN* amplification and the use of systemic therapy before, during, or after radiation, and *MYCN* amplification was not associated with the use of anti-GD2 antibody therapy; however, given the many different relapse regimens used, we were not able to further study whether specific systemic therapy regimens were selected to treat patients with *MYCN*-amplified disease. Future prospective studies with larger cohorts are needed to confirm these findings and explore whether other molecular factors may correlate with poor prognosis or in-field recurrence.

More patients who received hypo-RT had progression of disease prior to RT compared to those receiving conv-RT, suggesting that hypo-RT was likely used more often in patients with more rapidly progressive disease (Table 1). We attribute this difference to the time-sensitive demands of rapidly progressive disease; patients with disease progression before RT likely needed to begin therapy sooner, required additional lines of systemic therapy that necessitated shorter radiation treatments, and had decreased performance status that called for shorter dose-fractionation regimens. Furthermore, these data suggest that patients with more aggressive disease may have received hypo-RT; despite this bias against the hypo-RT group, we found comparable local control and symptomatic benefit after hypo-RT compared to conv-RT. Our results also complement recent results by Sudmeier et al., whose findings indicate that palliative RT courses delivered in fewer fractions are effective in controlling pain and are associated with limited toxicities in pediatric patients with cancer [12]. In our cohort, patients tolerated hypo-RT well and this approach appears to be safe as grade 2 or 3 toxicities were limited. There was no statistically significant difference between the two groups with regard to grade 2 or 3 toxicities. This is likely due to modest doses of radiation indicated for neuroblastoma.

Delivering palliative RT in fewer fractions offers children facing a terminal diagnosis the opportunity to spend fewer days in the hospital and more days at home without compromising palliation. Length of treatment course, treatment expense, and transportation can be barriers to receiving palliative radiotherapy in patients with metastatic disease [20]. Hypo-RT has also been shown to be more cost-effective than palliative chemotherapy or narcotic analgesics in certain patient populations [21]. Shorter radiation courses would also reduce the need for sedation and anesthesia in many young patients with high-risk neuroblastoma, which reduces uncommon complications of anesthesia and financial burdens and improves the quality of life, including limiting the need for fasting [20,22].

Limitations of our study include its retrospective design and relatively small cohort size. Patient selection for conv-RT and hypo-RT may also influence our results. However, given that more patients in the hypo-RT group had progression of disease prior to RT, patients in the hypo-RT group likely had more aggressive diseases and thus would bias the results toward worse outcomes in the hypo-RT group. Thus, these differences between the conv-RT and hypo-RT groups would unlikely change our conclusion of similar clinical outcomes between the two groups. While we defined hypo-RT as any treatment course with >2 Gy per fraction, both the conv-RT and hypo-RT groups comprised patients receiving various dose-fractionation regimens, which may affect the efficacy of radiation. Our analysis showed that local control did not differ between patients receiving BED ≥ 25.5 Gy compared to those receiving <25.5 Gy; however, further studies are needed to determine whether there is an optimal dose for palliation for relapsed/refractory high-risk neuroblastoma. The rates of toxicity may be underreported as more subtle toxicity may not always be documented in the medical records. The median follow-up for our cohort is relatively short; thus, long-term toxicity after hypo-RT could not be reported. In addition, given the retrospective nature of our study, we were not able to prospectively, and quantitatively assess symptomatic benefits from conv-RT and hypo-RT. Thus, future prospective studies are needed to better understand whether these approaches may result in a different time to clinical benefit or duration of symptomatic benefit and to better capture toxicities. There remain challenges to studying these questions in a prospective manner for rare cancers, such as neuroblastoma, and to achieving adequate long-term follow-up regarding the efficacy and safety of hypo-RT. Nonetheless, cooperative groups or consortia, such as Children’s Oncology Group, International Society of Paediatric Oncology, and New Approaches to Neuroblastoma Therapy (NANT), have conducted many successful trials for neuroblastoma and may present the best opportunity for radiotherapy and quality of life questions to be embedded in studies regarding novel systemic therapy approaches, particularly for patients with relapsed or refractory neuroblastoma.

In conclusion, our findings demonstrate the efficacy of hypo-RT for the palliation of bony and soft tissue metastases in patients with relapsed or refractory high-risk neuroblastoma. Hypo-RT also has the benefits of reducing the financial, logistical, and anesthetic burdens of longer treatment courses, and thus, should be considered when the quality of life from shorter radiotherapy courses is of benefit to patients and their families.

## Figures and Tables

**Table 1:** Clinical and treatment characteristics for the first course of palliative radiotherapy.

	Entire Cohort n (%)	Conv-RT n (%)	Hypo-RT n (%)	*p*-Value
Sex				0.508
Female	20 (54)	10 (63)	10 (48)	
Male	17 (46)	6 (38)	11 (52)	
Age at diagnosis				0.254
≤18 months	8 (22)	5 (31)	3 (14)	
>18 months	29 (78)	11 (69)	18 (86)	
Age at the first course of palliative RT				1.000
≤6 years	20 (54)	9 (56)	11 (52)	
>6 years	17 (46)	7 (44)	10 (48)	
Primary site				0.180
Adrenal/abdominal	35 (95)	14 (88)	21 (100)	
Other	2 (5)	2 (13)	0 (0)	
*MYCN* amplification				0.074
No	10 (27)	4 (25)	6 (29)	
Yes	16 (43)	10 (63)	6 (29)	
Unknown	11 (30)	2 (13)	9 (43)	
Unfavorable histology				0.380
No	1 (3)	1 (6)	0 (0)	
Yes	23 (62)	11 (69)	12 (57)	
Unknown	13 (35)	4 (25)	9 (43)	
Refractory disease				0.702
No	28 (76)	13 (81)	15 (71)	
Yes	9 (24)	3 (19)	6 (29)	
Progression of disease before palliative RT				0.017 *
No	24 (65)	14 (88)	10 (48)	
Yes	13 (35)	2 (13)	11 (52)	
Bony site for RT				0.254
No	8 (22)	5 (31)	3 (14)	
Yes	29 (78)	11 (69)	18 (86)	
RT type				0.794
2D	21 (57)	9 (56)	12 (57)	
3D	4 (11)	1 (6)	3 (14)	
IMRT/VMAT	9 (24)	4 (25)	5 (24)	
Protons	3 (8)	2 (13)	1 (5)	
Concurrent chemotherapy with palliative RT				0.705
No	29 (78)	12 (75)	17 (81)	
Yes	8 (22)	4 (25)	4 (19)	
Chemotherapy after palliative RT				0.207
No	6 (17)	1 (7)	5 (25)	
Yes	29 (83)	14 (93)	15 (75)	
Asymptomatic lesion				0.055
No	29 (78)	10 (63)	19 (91)	
Yes	8 (22)	6 (38)	2 (10)	
Clinical benefit after RT (Symptomatic relief or decrease in size of lesion)				1.000
No	3 (9)	1 (8)	2 (10)	
Yes	30 (91)	12 (92)	18 (90)	
Best imaging response after RT				0.304
Complete response	7 (24)	4 (29)	3 (20)	
Partial response	18 (62)	10 (71)	8 (53)	
Stable disease	2 (7)	0 (0)	2 (13)	
Progressive disease	2 (7)	0 (0)	2 (13)	
Biologically effective dose				0.475
<25.5 Gy	11 (30)	6 (38)	5 (24)	
≥25.5 Gy	26 (70)	10 (63)	16 (76)	
In-field recurrence				1.000
No	25 (68)	11 (69)	14 (67)	
Yes	12 (32)	5 (31)	7 (33)	

* *p* < 0.05.

**Table 2:** Univariate analyses for time to in-field progression with death as a competing risk.

	Univariate	
	SHR (95% CI)	*p*-Value
Sex		
Female	Ref.	
Male	0.74 (0.24–2.26)	0.599
Age at the first course of palliative RT		
≤6 years	Ref.	
>6 years	2.64 (0.79–8.76)	0.114
*MYCN* amplification		
No	Ref.	
Yes	0.21 (0.05–0.78)	0.021 *
Unknown	0.34 (0.08–1.47)	0.149
Refractory disease		
No	Ref.	
Yes	0.83 (0.23–2.99)	0.776
Progression of disease before palliative RT		
No	Ref.	
Yes	1.53 (0.48–4.85)	0.471
Bony site for RT		
No	Ref.	
Yes	0.52 (0.17–1.63)	0.262
Concurrent chemotherapy with palliative RT		
No	Ref.	
Yes	0.26 (0.04–1.91)	0.187
Chemotherapy after palliative RT		
No	Ref.	
Yes	2.04 (0.24–17.67)	0.517
Asymptomatic lesion		
No	Ref.	
Yes	0.31 (0.04–2.23)	0.246
Type of RT		
Conv-RT	Ref.	
Hypo-RT	1.17 (0.39–3.58)	0.778
BED		
<25.5 Gy	Ref.	
≥25.5 Gy	1.39 (0.38–5.05)	0.618

* *p* < 0.05.

**Table 3:** Clinical and treatment characteristics for all courses of palliative radiotherapy.

	Entire Cohort n (%)	Conv-RT n (%)	Hypo-RT n (%)	*p*-Value
Refractory disease				0.797
No	68 (76)	22 (73)	46 (77)	
Yes	22 (24)	8 (27)	14 (23)	
Progression of disease before palliative RT				0.056
No	63 (70)	25 (83)	38 (63)	
Yes	27 (30)	5 (17)	22 (37)	
Bony site for RT				0.083
No	25 (28)	12 (40)	13 (22)	
Yes	65 (72)	18 (60)	47 (78)	
RT type				0.089
2D	56 (64)	20 (67)	36 (62)	
3D	14 (16)	1 (3)	13 (22)	
IMRT/VMAT	15 (17)	7 (23)	8 (14)	
Protons	3 (3)	2 (7)	1 (2)	
Concurrent chemotherapy with palliative RT				1.000
No	70 (78)	23 (77)	47 (78)	
Yes	20 (22)	7 (23)	13 (22)	
Chemotherapy after palliative RT				0.056
No	27 (30)	5 (17)	22 (37)	
Yes	63 (70)	25 (83)	38 (63)	
Asymptomatic lesion				0.062
No	69 (77)	19 (63)	50 (83)	
Yes	21 (23)	11 (37)	10 (17)	
Clinical benefit after RT (Symptomatic relief or decrease in size of lesion)				0.716
No	10 (13)	2 (9)	8 (14)	
Yes	70 (88)	21 (91)	49 (86)	
Best imaging response after RT				0.220
Complete response	8 (13)	5 (21)	3 (8)	
Partial response	32 (36)	14 (58)	18 (49)	
Stable disease	11 (18)	2 (8)	9 (24)	
Progressive disease	10 (16)	3 (13)	7 (19)	
BED				0.005 *
<25.5 Gy	32 (36)	17 (57)	15 (25)	
≥25.5 Gy	58 (64)	13 (43)	45 (75)	
In-field recurrence				1.000
No	61 (68)	20 (67)	41(68)	
Yes	29 (32)	10 (33)	19 (32)	

* *p* < 0.05.

**Table 4:** Univariate analysis for time to in-field progression with death as a competing risk for all courses using cluster analysis.

	Univariate	
	SHR (95% CI)	*p*-Value
Refractory disease		
No	Ref.	
Yes	1.56 (0.65–3.74)	0.320
Progression of disease before palliative RT		0.800
No	Ref.	
Yes	1.10 (0.51–2.38)	
Bony site for RT		0.010 *
No	Ref.	
Yes	0.43 (0.23–0.82)	
Concurrent chemotherapy with palliative RT		0.660
No	Ref.	
Yes	0.80 (0.29–2.19)	
Chemotherapy after palliative RT		0.050
No	Ref.	
Yes	3.61 (0.99–13.03)	
Asymptomatic lesion		0.460
No	Ref.	
Yes	0.67 (0.23–1.95)	
Type of RT		0.760
Conv-RT	Ref.	
Hypo-RT	0.89 (0.40–1.96)	
BED		0.320
≤25.5 Gy	Ref.	
>25.5 Gy	1.52 (0.67–3.43)	

* *p* < 0.05.

## Data Availability

The data that support the findings of this study are available on request from the corresponding author. The data are not publicly available due to privacy or ethical restrictions.

## Supplementary Materials

The following supporting information can be downloaded at: https://www.mdpi.com/article/10.3390/curroncol32030124/s1, Figure S1: Progression survival after first course of palliative radiotherapy stratified by *MYCN* amplification status; Figure S2: Overall survival after first course of palliative radiotherapy stratified by *MYCN* amplification status; Table S1: Association between *MYCN* amplification and systemic therapy; Table S2: Rates of Grade ≥2 Toxicity Attributed to the Radiation Field; Table S3: Follow-up imaging of the lesion after treatment stratified by type of lesion from all treatment courses.

## Abbreviations

COG	Children’s Oncology Group
RT	Radiotherapy
Conv-RT	Conventionally fractionated radiotherapy
Hypo-RT	Hypofractionated radiotherapy
BED	Biologically effective dose
