# Supplementary material for: Hypofractionated Palliative Radiotherapy for Relapsed and Refractory High-Risk Neuroblastoma
Source: Curr Oncol. 2025 Feb 22;32(3):124. doi: 10.3390/curroncol32030124 (PMC11941608; doi:10.3390/curroncol32030124)
Supplement: Supplementary file 1 [file curroncol-32-00124-s001.zip › curroncol-3475739-supplementary.pdf]

Supplemental Figure S1. Progression survival after first course of palliative radiotherapy stratified by *MYCN* amplification status.

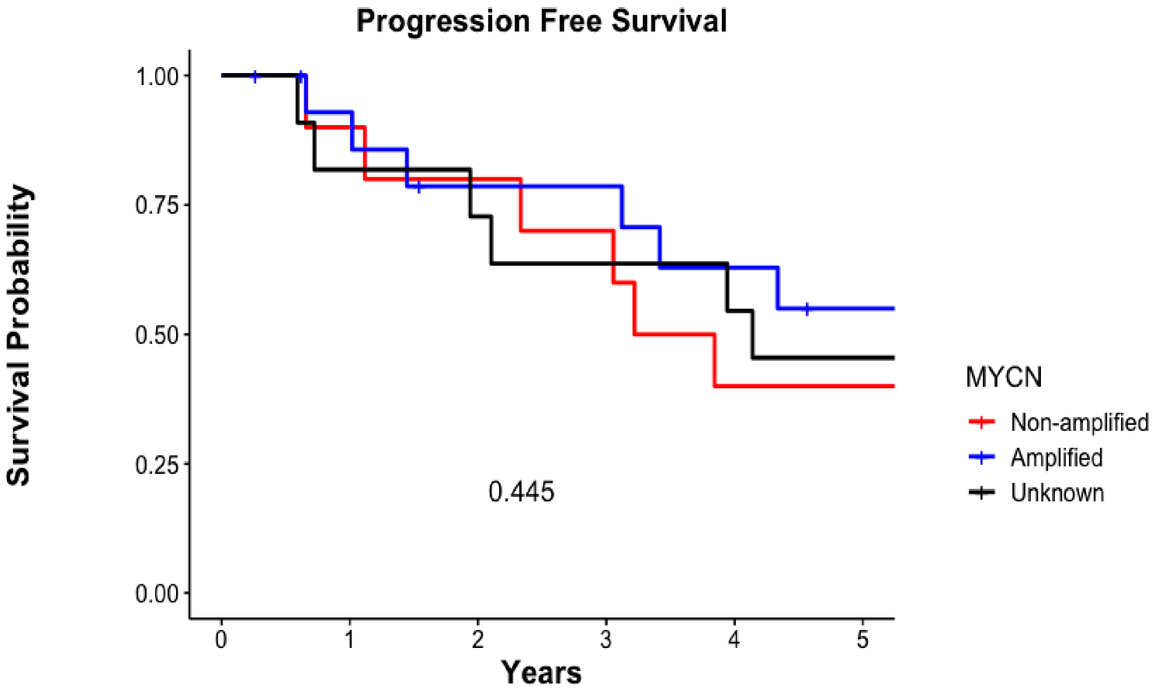

Number at risk

	0	1	2	3	4	5
Non-amplified	10	9	8	7	4	4
Amplified	16	13	10	10	8	6
Unknown	11	9	8	7	6	5

Supplemental Figure S2. Overall survival after first course of palliative radiotherapy stratified by *MYCN* amplification status.

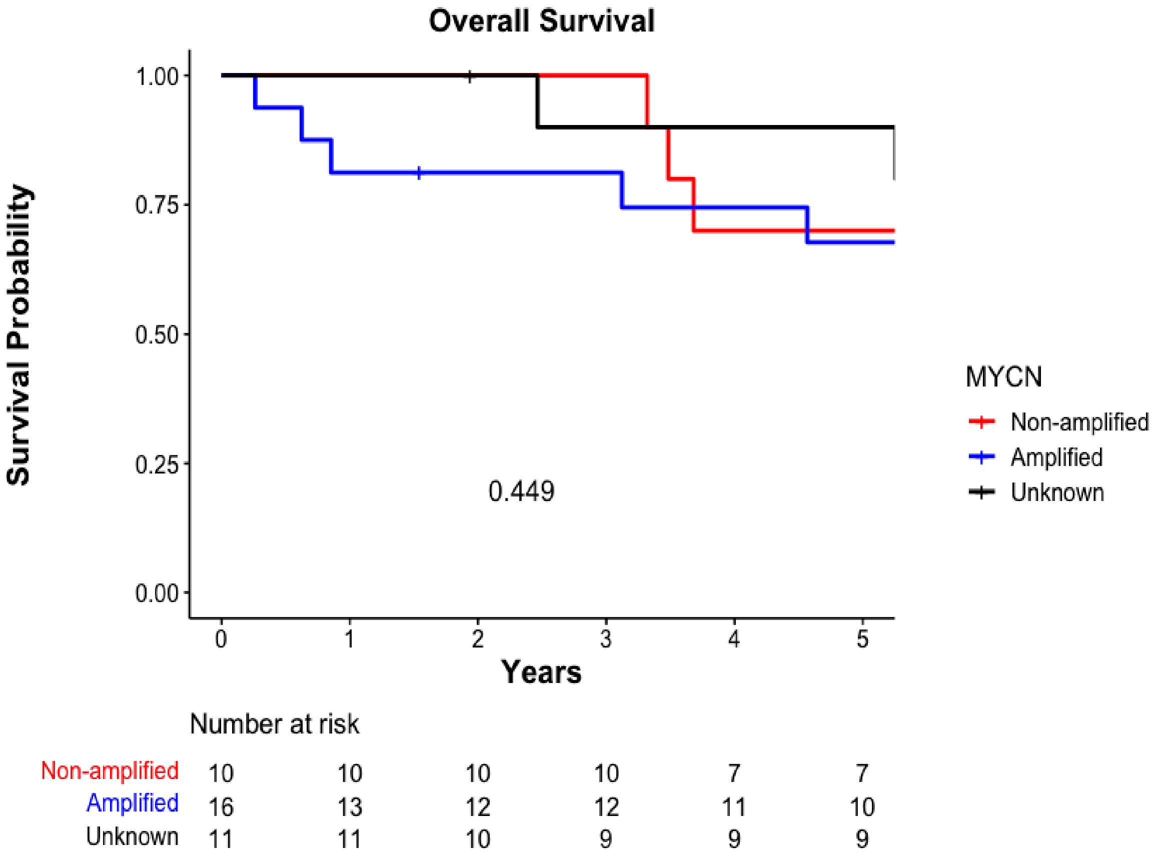

Supplemental Table S1. Association between *MYCN* amplification and systemic therapy.

	Pre-RT systemic therapy	Systemic therapy concurrent with RT	Post-RT systemic therapy	Regimens with Anti-GD2 antibody
MYCN amplification				
No (n = 10)	8 (80%)	2 (20%)	7 (70%)	2 (20%)
Yes (n = 16)	8 (50%)	5 (31%)	12 (75%)	5 (31%)
Unknown (n = 11)	9 (82%)	1 (9%)	10 (91%)	4 (36%)
P-value	0.178	0.416	0.602	0.815

Supplemental Table S2. Rates of Grade ≥2 Toxicity Attributed to the Radiation Field

	Conv-RT (n = 30)	Hypo-RT (n = 60)	p-value
Grade 2 Toxicity	3 (10%)	3 (5%)	0.40
Esophagitis	2 (7%)	2 (3%)	
Diarrhea	1 (3%)		
Mucositis		1 (2%)	
Grade 3 Toxicity	3 (10%)	2 (3%)	0.33
Mucositis	2 (7%)	2 (3%)	
Sepsis	1 (3%)		

Supplemental Table S3. Follow-up imaging of the lesion after treatment stratified by type of lesion from all treatment courses

	Bony Site for RT		p-value
	No (n = 25)	Yes (n = 65)	
MRI	Median: 0 (range: 0-2)	Median: 0 (range:0-20)	0.121 #
PET/CT	Median: 0 (range: 0-2)	Median: 0 (range: 0-2)	0.898 #
MIBG	Median: 1 (range: 0-12)	Median: 1 (range: 0-19)	0.742 #
CT	Median: 2 (range: 0-13)	Median: 0 (range: 0-10)	<0.0001 #*
No imaging§	4 (16%)	16 (25%)	0.572 +
CT-based imaging only	4 (16%)	3 (5%)	0.090 +

#Mann-Whitney test
+Fisher exact test
*p<0.05
§Lesions without imaging were due to transition to end of life care and comfort measures for those patients
